# Privacy awareness among healthcare professionals in intensive care unit: A multicenter, cross-sectional study

**DOI:** 10.1097/MD.0000000000032930

**Published:** 2023-02-10

**Authors:** Ahmet Ozdinc, Zuleyha Aydin, Muhittin Calim, Ahmet Selim Ozkan, Huseyin Bakir, Sedat Akbas

**Affiliations:** a Department of Medical History and Ethics, Istanbul University-Cerrahpasa Cerrahpasa Faculty of Medicine, Istanbul, Turkey; b Department of Biostatistics and Medical Informatics, Istanbul University-Cerrahpasa Cerrahpasa Faculty of Medicine, Istanbul, Turkey; c Department of Anesthesiology and Reanimation, Bezmialem Vakif University School of Medicine, Istanbul, Turkey; d Department of Anesthesiology and Reanimation, Inonu University School of Medicine, Malatya, Turkey; e Clinic of Surgical Oncology, Samsun Training and Research Hospital, Samsun, Turkey; f Department of Anesthesiology and Reanimation, Bezmialem Vakif University School of Medicine, Istanbul, Turkey.

**Keywords:** healthcare professionals, in-service training, intensive care units, nurses, physicians, privacy

## Abstract

This multicenter, cross-sectional study aimed to determine and examine the privacy awareness and patient rights education of healthcare professionals working in intensive care units (ICUs). The primary purpose of this study was to determine the privacy awareness of healthcare professionals working in the ICU. In addition, the secondary aim was to examine the relationship between patient rights education and awareness scores, as well as to question the need for privacy awareness education. The study population consisted of ICU physicians, nurses, and allied health personnel working in university hospitals, training and research hospitals, state hospitals, and private hospitals in Turkey. The data were collected through a questionnaire prepared by the researchers, including a question set about sociodemographics, a question about patient rights education, and the privacy awareness scale (PAS) scores using online Google Forms. In the results of the study conducted among 569 participants, the mean total PAS score was 38.31 ± 2.54. The PAS score was significantly different according to the occupation. The PAS scores of the nurses were higher than physicians and allied health personnel. The PAS scores differed according to whether the participants had received patient rights education. This study found that nurses were the group with the highest PAS scores among healthcare professionals. In addition, the PAS scores of nurses working in private and training and research hospitals were higher than those of other hospital employees. On the other hand, the lowest scores belonged to university hospitals and receiving patient rights education increased the PAS score of the nurses. This study showed that all enrolled healthcare professionals required in-service training to gain privacy awareness.

## 1. Introduction

Privacy is understood as a basic human need^[1]^ and has been particularly emphasized in the fields of healthcare ethics and patient rights declarations.^[2–5]^ However, there is no generally accepted definition of the concept of privacy, despite its importance being constantly highlighted and it becoming one of the key principles of medical ethics, and many authors have noted the difficulties associated with defining this concept.^[6,7]^

The concept of privacy can be defined in various dimensions. Burgoon, a professor of communication, considers privacy in terms of its physical, psychological, social, and informational dimensions.^[8]^ Physical privacy is the degree to which a person is physically accessible to others. This access is related to personal space, which is a controversial concept. The American psychologist Sommer defines personal space as the invisible space that surrounds the human body and separates 1 person from others.^[9]^ Psychological privacy involves the rights of individuals to control their cognitive and emotional affects and influence, to form their own values, and under what conditions to share their thoughts and private information. In this context, privacy has also been considered freedom.^[10]^ Social privacy encompasses people ability and effort to control their social relationships. In this respect, privacy is closely related to culture, and culture has an essential effect on protecting people privacy.^[11]^ Finally, information privacy entails a person right to determine whether their personal data is disclosed to another person or organization. In this context, anonymity, which Westin defines as one of the states of privacy, will emerge when the individual is in public places or performs public actions but still seeks out and finds the freedom of identity and surveillance.^[12]^ These concepts are all valid for both practical and research issues.^[13]^

One privacy can be lost in 2 ways. One can either give up their privacy or it can be taken away.^[14]^ If 1 makes 1 matters public or exposes oneself, 1 gives up their privacy. This way is a self-responsible loss of control and is typically harmless. On the other hand, losing privacy against 1 will is often disturbing. Hospitals and other health institutions are places where a patient capacity to control the environment is limited, and there is often a loss of privacy.^[15]^ Physical, social, and informational privacy becomes important in such places where a person loses control.

One of the basic principles of modern medical ethics is respect for autonomy. Autonomy is “at least 1 say and power over oneself, without being under the control of others and without being in a situation that prevents them from making meaningful choices.”^[2]^ On the other hand, the principle of respect for autonomy requires the showing of a respectful attitude toward autonomous objects and taking action to show this respect. The principle of respect for autonomy in health services is especially effective among patients and health workers. In this context, the concepts discussed include honesty, confidentiality, loyalty, and privacy. When the word “privacy, defined as “confidentiality in the dictionary of the Turkish Language Institution, is seen as a right in health services, it is considered to be the “right to privacy.”^[16]^ The right to privacy is related to the individual fundamental rights, such as the right to life, freedom, and property.^[17]^ Therefore, professional secrecy must be strictly observed to protect this fundamental right in specific professions such as those in healthcare, as professional confidentiality includes the requirement that a person’s information not be disclosed in transactions related to professional duties.^[18]^

Privacy awareness, which can be defined as the degree of consciousness regarding privacy for oneself and others, is expected to be high in healthcare professionals. This consciousness develops based on 1 convictions, family values, as well as religious and social judgments.^[19]^ With this objective in mind, a variety of scales have been developed to measure the general impact of the environment, direct privacy education, and enhanced privacy awareness.^[20–26]^ Of these scales, we have used the privacy awareness scale (PAS), which originated in Japan; the Turkish version was developed in 2019. The reason for using this scale is that it has both validity and reliability compared to other scales and has been applied specifically to healthcare professionals and students.

## 2. Materials and methods

### 2.1. Study hypothesis

The environments of operating rooms and intensive care units (ICUs) come first among places in which privacy loss is most common in hospitals. The length of a patient stay in the ICU is approximately 4 to 5 days.^[27]^ Intensive care patients are exposed to dramatic life changes as they enter to ICU in the face of a sudden situation. Patients may experience fear, loneliness, and isolation in such an unusual situation.^[28]^ These feelings thus make it difficult for patients to protect their privacy.

On the other hand, healthcare professionals working in ICUs have been shown to be under more stress and experience more time constraints due to limited resources and organizational difficulties than those working in other healthcare settings.^[29]^ For these reasons, ICUs differ from other units or services in hospitals. Therefore, in this study, we selected our sample from ICUs while considering this difference.

### 2.2. Study purpose

The primary purpose of this study was to determine the privacy awareness of healthcare professionals working in the ICU. In addition, the secondary aim was to examine the relationship between patient rights education and awareness scores, as well as to question the need for privacy awareness education.

### 2.3. Significance of the study

The study is important in examining the privacy awareness and related factors of ICU physicians, nurses, and allied health personnel (AHPs) working in ICUs providing critical patient care for the first time. In addition, this study stands out as it was first carried out in more than 20 various hospitals across Turkey.

### 2.4. Ethics committee approval

This study was approved by Istanbul University–Cerrahpasa Clinical Research Ethics Committee (protocol no: 419909; date: 10.05.2022). Informed consent was obtained from the participants for publication of this clinical trial details.

### 2.5. Study population and sample size

The study population consisted of ICU physicians, nurses, and AHPs working in the university hospitals, training and research hospitals, state hospitals, and private hospitals in Turkey between June and July 2022. The minimum sample size was calculated as 516 with an effect size of 0.137, alpha value of 0.05, and power of 0.80 using the “G. Power-3.1.9.7” program. We ultimately included a total of 569 people to allow for drop-out. post hoc analysis was conducted to determine the study’s strength, and 1-way analysis of variance (ANOVA) showed that the effect size was 0.198; the alpha value was 0.05, and the power of the study (1-β) was 0.99.

### 2.6. Data collection tools

The data were collected through a questionnaire prepared by the researchers including a question set about socio-demographics, a question about patient rights education, and the PAS scores.

### 2.7. Privacy awareness scale

**Figure 1.**
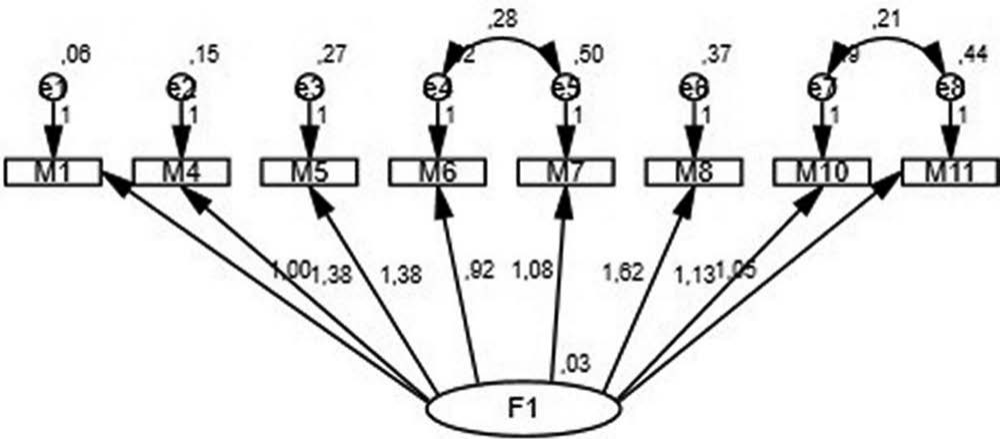
Confirmatory factor analysis model of the PAS. PAS = privacy awareness scale.

This is a scale developed by Tabata and Hirotsune in 2014 to measure privacy awareness.^[30]^ The scale consists of 15 items and 3 factors. The first factor is the awareness of privacy for oneself, the second factor is the awareness of privacy for others, and the third factor is the behavior of maintaining the privacy of others. The scale was prepared using a 5-point Likert format (1 – Strongly disagree, 5 – Strongly agree), translated into the Turkish language by Öztürk et al^[31]^ in 2019 and a 3-factor structure preserved (F1 [M1, M2, M3, M4], F2 [M5, M6, M7, M8], and F3 [M9, M10, M11]. There were 11 items included in the scale. In this study, reverse items were determined as M6, M7, M10, and M11, and reverse coding was performed. The Cronbach’s alpha reliability coefficient was lower than the original scale. In order to increase this value, confirmatory factor analysis was performed, and factor loadings were examined. As a result of the analysis, M2, M3, and M9 were removed, the remaining items were collected under a single factor, and the analysis continued. The confirmatory factor analysis model is presented in Figure 1, and the fit indices of the model are CMIN/DF (2.134), RMSEA (0.045), GFI (0.983), AGFI (0.967), NFI (0.937), and CFI (0.965), with values that could be considered good or acceptable.^[32]^ The Cronbach’s alpha coefficient of the study was found to be 0.589, and the scale total score was between 0 and 40.

### 2.8. Data collection method

The questionnaire form created for the study was sent to healthcare professionals online using Google Forms. However, to ensure the confidentiality of the data in the study, no personally identifiable information was requested, and a commitment was made to the voluntary participants regarding data privacy.

### 2.9. Limitations of the study

This study had some limitations. First, the study was conducted in an electronic form. Second, the study was carried out only with healthcare professionals in the ICUs in Turkey; therefore, the study results may not globally apply to different regions or countries. Third, we applied all questions to healthcare professionals who worked in adult ICUs, not pediatric. Thus, we predict that the results for pediatric ICUs will be different.

### 2.10. Statistical analysis

Data were analyzed using the SPSS program version 22.0 for Windows (Statistical Package for the Social Sciences, IBM Corp., Armonk, NY). Quantitative data are presented as mean or standard deviation, and categorical data are shown as numbers or percentages. Since the sample size was greater than 50, the Kolmogorov–Smirnov normality test was used to determine whether quantitative variables showed a normal distribution. Since the results were *P*< .05, the Skewness–Kurtosis values were examined, and the variables in the range of ±3.29 were considered to be normally distributed.^[33]^ An independent 2-sample *t* test was used to compare the normally distributed 2-group variables, and a chi-square test was used to compare categorical variables. Variables involving 3 or more groups were also analyzed with 1-way ANOVA if they were normally distributed and with the Kruskal–Wallis test if they did not show normal distribution. The homogeneity of the variances was checked with Levene’s test statistic, and in the case of inhomogeneity (*P* < .05), Welch’s test was used as an alternative method instead of the *F* test. As a result of the analysis, the difference between the groups was found to be significant, and the comparison between the groups in the variables with normal distribution was analyzed using the Tamhane test. Variables not showing normal distribution were evaluated using the Bonferroni test.

## 3. Results

Descriptive statistics of the socio-demographic characteristics of healthcare professionals working in the ICU are presented in Table 1. Of the 569 healthcare professionals who participated in the questionnaire, there were 348 (61.2%) nurses, with 334 (58.7%) being women. The highest number of individuals were in the 20 to 29-years-old age range, with 275 (48.3%) of the 569 healthcare professionals belonging to this demographic. There were 181 (31.8%) participants graduated from medical school, with this being the largest graduation category; 180 (31.6%) of the participants worked in private hospitals, and most participants had been working between 0 and 5 years, with 249 (43.8%) individuals when the total working years in the occupation are examined. Considering the participants years of work in the ICU, 290 (51%) had worked between 0 and 4 years. We concluded that 420 (73.8%) of the healthcare professionals who participated in the questionnaire had received patient rights education, while 149 (26.2%) had not.

**Table 1:** Descriptive statistics of the socio-demographics of healthcare professionals.

Variables		n	%
Occupation	Physician	181	31.8
	Nurse	348	61.2
	AHP	40	7
Gender	Female	334	58.7
	Male	235	41.3
Age	20-29	275	48.3
	30-39	161	28.3
	40 and above	133	23.4
Graduation	High school	143	25.1
	Associate degree	71	12.5
	Bachelor’s degree (for nurses)	151	26.5
	Master	23	4
	Medical faculty	181	31.8
Institution	University hospital	120	21.1
	Training and research hospital	167	29.3
	Public hospital	102	17.9
	Private hospital	180	31.6
Working years in the occupation	0–5	249	43.8
	6–11	146	25.7
	12–17	87	15.3
	18 and above	87	15.3
Working years in the ICU	0–4	290	51
	5–9	136	23.9
	10–14	74	13
	15 and above	69	12.1
Patient rights education	Received	420	73.8
	Did not received	149	26.2

% = percent, AHP = allied health personnel, ICU = intensive care unit, n = number.

Table 2 shows that the mean total PAS score was 38.31 ± 2.54, with the participants scoring between 28 and 40. In terms of the PAS, the Skewness–Kurtosis values were examined. The skewness value was -1622, the kurtosis value was 1871, and the total PAS score was considered to be normally distributed.

**Table 2:** The mean total PAS score.

Variable	n	Min	Max	Mean	SD	Skewness	Kurtosis
Total PAS score	569	28	40	38,31	2543	−1622	1871

Max = maximum, Min = minimum, n = number, PAS = privacy awareness scale, SD = standard deviation.

A comparison of the patient rights education of healthcare professionals with their occupation, age, gender, graduation level, institution, working years in the occupation, and working years in the ICU was analyzed using a chi-square test (Table 3). A significant relationship was found between the occupations of healthcare professionals and their status in terms of receiving patient rights education (X^2^_(2)_ = 120.598, *P* < .05). The ratio of nurses and AHPs who received patient rights education was higher than those who did not. The ratio of physicians who did not receive patient rights education was higher than those who did. A significant relationship was found between age groups and the status of receiving patient rights education (X^2^_(2)_ = 17.409, *P* < .05). The ratio of receiving patient rights education in all age groups was found to be higher than those who did not. A significant relationship was found between gender and the status of receiving patient rights education (X^2^_(1)_ = 6.797, *P* < .05). It was determined that the ratio of those who received education was higher than those who did not receive education in both gender groups. A significant relationship was found between education level and patient rights education (X^2^_(4)_ = 121.654, *P* < .05). The ratio of individuals who had received patient rights in high school, associate degree, Bachelor’s degree, and master graduate was higher than those who had not. On the other hand, we found that the ratio of those who had not received patient rights education was higher in those graduated from medical school. A significant relationship was found between the institution of employment and the status of receiving patient rights education (X^2^_(3)_ = 41.776, *P* < .05). We found that the ratio of those who had received education in all institutions was higher than those who had not. There was no statistically significant relationship between the variables of working years in the occupation and working years in the ICU as well as the status of receiving patient rights education (*P* > .05).

**Table 3:** A comparison of the patient rights education of healthcare professionals with variables.

Variables		Patient rights education				χ^2^	*P* value
		Received		Did not received			
		n	%	n	%		
Occupation	Physician	80	44.2	101	55.8	120.598	< .001*
	Nurse	306	87.9	42	12.1		
	AHP	34	85.0	6	15.0		
Age	20–29	224	81.5	51	18.5	17.409	< .001*
	30–39	103	64.0	58	36.0		
	40+	93	69.9	40	30.1		
Gender	Female	260	77.8	74	22.2	6.797	.009*
	Male	160	68.1	75	31.9		
Graduation	High school	129	90.2	14	9.8	121.654	< .001*
	Associate degree	63	88.7	8	11.3		
	Bachelor’s degree (for nurses)	129	85.4	22	14.6		
	Master	19	82.6	4	17.4		
	Medical faculty	80	44.2	101	55.8		
Institution	University hospital	70	58.3	50	41.7	41.776	< .001*
	Training and research hospital	109	65.3	58	34.7		
	Public hospital	84	82.4	18	17.6		
	Private hospital	157	87.2	23	12.8		
Working yr in the occupation	0–5	193	77.5	56	22.5	5.886	.118
	6–11	108	74.0	38	26.0		
	12–17	56	64.4	31	35.6		
	18+	63	72.4	24	27.6		
Working yr in the ICU	0–4	214	73.8	76	26.2	2.819	.420
	5–9	96	70.6	40	29.4		
	10–14	60	81.1	14	18.9		
	15+	50	72.5	19	27.5		

% = percent AHP = allied health personnel, ICU = intensive care unit, n = number, χ2 **=** Kruskal Wallis *H* test.

* *P*< .05 is considered statistically significant.

Table 4 Compares the PAS to occupation, age, in-service training, graduation level, institution, working years in the ICU, gender, and patient rights education. This information was analyzed using 1-way ANOVA to compare the occupational group of healthcare professionals, need for in-service privacy education, and age variables in terms of PAS scores. Instead of 1-way ANOVA, the Welch’s test was used to interpret the variables not providing homogeneity (*P* < .05). The PAS score was shown to differ according to occupation (Welch’s test_(2,98)_ = 10.018, *P* < .05). We observed that the scores of the nurses were higher than those of the physicians. The difference between the results of the question regarding the need for in-service privacy education and PAS was found to be statistically significant (Welch’s test_(4,38.42)_ = 3.486, *P* < .05). Those who chose the “strongly agree” option were shown to have higher scores than those who chose the “I agree” option. PAS was shown not to differ according to age (*P* > .05). The comparison of PAS according to the healthcare professional graduation level, institution, occupation, and working years in the ICU was analyzed using the Kruskal–Wallis *H* test. The PAS scores of individuals differed according to their graduation levels (Kruskal Wallis H test [χ^2^] = 23.856, *P* < .05). The PAS scores of high school graduates and Bachelor’s degree has been found to be higher than those graduated from medical school. In the present work, the PAS scores analysis was found to be statistically significant according to the institutions (χ^2^ = 19.750, *P* < .05). The PAS scores of employees in the training and research hospitals was observed to be higher than that of employees in the university hospitals, and the PAS scores of employees in the private hospital was found to be higher than that of the employees in the state and university hospitals. The PAS was found not to differ according to the working years in the occupation and in the ICU (*P* > .05). A comparison of the PAS scores of healthcare professionals according to gender and whether they have received patient rights education was analyzed using a 2-sample *t* test. The analyses showed that PAS did not differ according to gender (*P* > .05). The PAS was found to differ according to whether the participants had received patient rights education (*t*_(567)_ = 2.967, *P* < .05). Those who had received training were found to have higher privacy awareness.

**Table 4:** Comparing the PAS scores to occupation, age, in-service training, graduation level, institution, working years in ICU, gender, and patient rights education.

Variables		n	Mean	SD	F	*P* value	Significant difference
Occupation	Physician	181	37.65	2.86	10.018_(W)_	< .001*	2 > 1
	Nurse	348	38.71	2.19			
	AHP	40	37.88	3.21			
In-service privacy training is required	Strongly disagree	18	38.28	2.47	3.486_(W)_	.016*	5 > 4
	Disagree	10	37.3	2.67			
	Undecided	39	37.77	3.26			
	Agree	44	36.93	3.02			
	Strongly agree	458	38.52	2.38			
Age	20–29	275	38.55	2.3	2.515	.082	
	30–39	161	38.02	2.74			
	40+	133	38.17	2.74			
		n	Mean rank	SD	χ^2^	P	Significant difference
Graduation	High school	143	317.13	4	23.856	< .001*	
	Associate degree	71	275.53				1 > 5
	Bachelor’s degree (for nurses)	151	299.78				3 > 5
	Master	23	335.78				
	Medical faculty	181	244.55				
Institution	University hospital	120	244.30	3	19.750	< .001*	2 > 1
	Training and research hospital	167	298.51				4 > 1
	Public hospital	102	259.35				4 > 3
	Private hospital	180	314.13				
Working years in the occupation	0–5	249	281.53	3	4.688	.196	
	6–11	146	288.36				
	12–17	87	263.05				
	18+	87	311.26				
Working years in the ICU	0–4	290	280.28	3	0.849	.838	
	5–9	136	294.48				
	10–14	74	283.59				
	15+	69	287.64				
		n	mean	SD	t	P	
Gender	Female	334	38.28	2.52	−0.384	.701	
	Male	235	38.36	2.58			
Patient rights education	Received	420	38.50	2.41	2.967	.003*	
	Did not received	149	37.79	2.84			

% = percent, AHP = allied health personnel, F = one-way ANOVA, ICU = intensive care unit, n = number, PAS = privacy awareness scale, SD = standard deviation, *t* = Independent *t* test, W = Welch’s test, χ^2^ = Kruskal Wallis H test.

* *P*<.05 is considered statistically significant,

For a total of 181 physicians, as shown in Table 5, the age, institution, working years in the ICU, occupation, gender, and patient rights education status were analyzed in terms of privacy awareness. First, the relationship between the PAS scores and age, institution, and working years in the ICU was analyzed with using a 1-way ANOVA test. We observed that the PAS scores in physicians did not differ according to age, institution, or years working in the ICU (*P* > .05). The comparison of the PAS scores of physicians with the working years in a given occupation was analyzed using the Kruskal–Wallis *H* test. The PAS scores of the physicians were found not to differ according to the working years in the indicated occupation (*P* > .05). Finally, the comparison of the PAS scores of physicians with gender and patient rights education was analyzed using a 2-sample *t* test. The analysis showed that the PAS of the physicians did not differ according to gender and patient rights education (*P* > .05).

**Table 5:** Comparison of the variables with the PAS scores for physicians.

Variables		n	Mean	SD	F	*P* value
Age	20–29	22	36.82	3.14	1.858	.159
	30–39	89	37.52	2.82		
	40+	70	38.09	2.78		
Institution	University Hospital	60	37.53	2.81	1.130	.338
	Training and research hospital	86	37.95	2.83		
	Public hospital	22	36.73	3.07		
	Private hospital	13	37.77	2.83		
Working years in the ICU	0–4	69	37.42	2.97	0.326	.806
	5–9	44	37.64	2.80		
	10–14	31	37.97	2.99		
	15+	37	37.84	2.66		
		n	Mean rank	SD	χ^2^	P
Working years in the occupation	0–5	36	79.47	3	6.687	.083
	6–11	47	86.94			
	12–17	52	88.89			
	18+	46	106.55			
		n	Mean	SD	t	P
Gender	Female	83	37.31	2.98	−1.472	.143
	Male	98	37.94	2.74		
Patient rights education	Received	80	37.63	2.87	−0.112	.911
	Did not received	101	37.67	2.86		

F = one-way ANOVA, ICU = intensive care unit, n = number, PAS = Privacy Awareness Scale, SD = standard deviation, t = Independent *t* test %percent, χ^2^ = Kruskal Wallis *H* test.

* *P*<.05 is considered statistically significant.

For 348 nurses, as shown in Table 6, PAS was analyzed in terms of graduation level, institution, age, working years in the ICU, occupation, gender, and patient rights education. This analysis was done using the Kruskal–Wallis *H* test. The PAS scores of nurses was shown to differ significantly according to graduation level (χ ^2^ = 9.094, *P* < .05). The PAS scores of high school graduates were significantly higher than those of associate-degree graduates. The PAS scores of nurses significantly differed according to the institution of employment (χ^2^ = 16.979, *P* < .05). The PAS scores of nurses working in private hospitals was observed to be higher than those working in university hospitals. The PAS scores of the employees of the training and research hospitals was higher than those of the employees of the university hospital. The PAS scores of nurses was found not to differ according to age, occupation, and working years in the ICU (*P* > .05). The relationship between the PAS scores of nurses with gender and patient rights education was analyzed using a 2-sample *t* test. The analysis showed that the PAS scores did not differ according to gender (*P* > .05). We found that there was a significant difference between the PAS scores and the status of receiving patient rights education (*t* [346] = 2.093, *P* < .05). Furthermore, educated individuals were observed to have higher PAS scores than those without such education.

**Table 6:** Comparison of the variables with the PAS scores for nurses.

		n	Mean rank	SD	χ^2^	p	significant difference
Graduation	High school	109	191.22	3	9.094	**0.028** *	1>2
	Associate degree	66	152.93				
	Bachelor's degree (for nurses)	150	169.29				
	Master	23	191.11				
Institution	University hospital	43	131.79	3	16.979	**0.001** *	4>1
	Training and research hospital	77	191.94				2>1
	Public hospital	73	159.75				
	Private hospital	155	184.64				
Age	20–29	241	172.54	2	0.816	.665	
	30–39	64	174.29				
	40+	43	185.80				
Working years in the occupation	0–5	191	165.82	3	5.421	.143	
	6–11	88	183.47				
	12–17	31	173.61				
	18+	38	198.11				
Working years in the ICU	0–4	198	165.94	3	4.866	.182	
	5–9	83	187.78				
	10–14	38	176.7				
	15+	29	192.09				
		n	Mean	SD	t	P	
Gender	Female	227	38.63	2.22	−0.847	0.398	
	Male	121	38.84	2.12			
Patient rights education	Received	306	38.8	2.09	2.093	.037*	
	Did not received	42	38.05	2.73			

% = percent, ICU = intensive care unit, n = number, PAS = Privacy Awareness Scale, SD = standard deviation, t = Independent *t* test, χ^2^ = Kruskal Wallis *H* test.

* *P*<.05 is considered statistically significant.

The participants responses to the question of whether there is a need for in-service privacy training according to the variables are examined in Table 7. Regarding the in-service training requirements, 88.2% of all participants gave a positive answer, and 5% gave a negative answer. In terms of the occupation variable, 154 (85.1%) of the physicians, 315 (90.5%) of the nurses, and 33 (82.5%) of the AHPs gave positive answers (strongly agree, agree). Regarding the age variable, 249 (90.5%) of those aged 20 to 29 years, 141 (87.6%) of those aged 30 to 39 years, and 112 (84.2%) of those aged 40 years and older answered “agree.” In terms of the gender variable, 299 (89.5%) of the women and 203 (86.4%) of the men answered positively (strongly agree, agree). With regard to the graduation level variable, 123 (86%) of the high school graduates, 67 (94.4%) of the associate degree graduates, 136 (90.1%) of the Bachelor’s degree graduates, 22 (95.7%) of the graduate school graduates, and 154 (85.1%) of the medical school graduates positive answered positively (strongly agree, agree). In terms of the institution variable, 105 (87.5%) of those working in a university hospital, 142 (85%) of those working in a training and research hospitals, 90 (88.2%) of those working in a public hospital, and 165 (91.7%) of those working in a private hospital answered positively (strongly agree, agree). Regarding the variable of the working years in a given occupation, 220 (88.4%) of the employees had between zero and 5 years of work experience, 134 (91.8%) had between 6 and 11 years, and 73 (83.9%) of had between 12 and 17 years; 75 (86.2%) of those with 18 years answered positively (strongly agree, agree). In terms of the variable for the working years in the ICU, 257 (88.7%) had worked between 0 and 4 years, 120 (88.2%) had worked between 5 and 9 years, and 64 (86.5%) had worked between 10 and 14 years; 61 (88.4%) of those with 15 years or more of experience answered positively (strongly agree, agree).

**Table 7:** Descriptive statistics on the need for in-service privacy training.

		The need for in-service privacy training					
		Negative data (strongly agree, agree)		Undecided		Positive data (strongly agree, agree)	
		n	%	n	%	n	%
Occupation	Physician	11	6.1	16	8.8	154	85.1
	Nurse	15	4.3	18	5.2	315	90.5
	AHP	2	5	5	12.5	33	82.5
Age	20-29	12	4.4	14	5.1	249	90.5
	30-39	12	7.5	8	5	141	87.6
	40+	4	3.1	17	12.8	112	84.2
Gender	Female	11	3.3	24	7.2	299	89.5
	Male	17	7.2	15	6.4	203	86.4
Graduation	High school	11	7.7	9	6.3	123	86
	Associate degree	1	1.4	3	4.2	67	94.4
	Bachelor’s degree (for nurses)	5	3.3	10	6.6	136	90.1
	Master	0	0	1	4.3	22	95.6
	Medical faculty	11	6.1	16	8.8	154	85.1
Institution	University hospital	6	5	9	7.5	105	87.5
	Training and research hospital	8	4.8	17	10.2	142	85
	Public hospital	6	5.9	6	5.9	90	88.2
	Private hospital	8	4.5	7	3.9	165	91.6
Working years in the occupation	0**–**5	11	4.4	18	7.2	220	88.3
	6**–**11	9	6.2	3	2.1	134	91.7
	12**–**17	6	6.9	8	9.2	73	83.9
	18 and above	2	2.3	10	11.5	75	86.2
Working years in the ICU	0**–**4	13	4.5	20	6.9	257	88.7
	5**–**9	10	7.3	6	4.4	120	88.2
	10**–**14	4	5.5	6	8.1	64	86.5
	15 and above	1	1.4	7	10.1	61	88.4
Patient rights education	Received	21	5	25	6	374	89
	Did not received	7	4.7	14	9.4	128	86
All Participants		28	5	39	6.9	502	88.2

% = percent, AHP = allied health personnel, ICU = intensive care unit, n = number.

## 4. Discussion

### 4.1. The PAS score and factors affecting this score

In this study conducted among 569 participants, the PAS score was 38.31 out of 40 on average. This score can be considered high in general. In the literature, similar results have been found in studies using the same or similar scales.^[23,31,34]^ According to the study, the group with the highest PAS score was nurses. This remarkable result had a statistically significant difference in favor of the nurses in the nurse-physician comparison.

In the literature review, no studies compare physicians and nurses regarding their awareness of privacy. Only physician-nurse differences, difficulties in decisions-making, and attitudes toward patients end-of-life care were discussed.^[35,36]^ Possible reasons for the differences between physicians and nurses may be that nurses are more likely to empathize with patients because they spend more time with them.^[37]^ The positive effects of empathy and professional values on ethical decision-making has been proven by some studies.^[38,39]^ The nurse-patient relationship is crucial because nurses create a strong healthcare workforce and have more patient contact time. In addition, given that the duties of nurses do not involve directly preparing treatment, it can be assumed that nurses will have a stronger social relationship with patients, as they are more likely to care for patients, administer treatments, and create safe and healthy environments.^[40]^

In our study, 3 factors affecting the PAS score of nurses were determined. The first is the institution in which they work, the second is whether they have received patient rights education, and the third is their level of graduation.

Private hospitals and training and research hospitals were determined to be among the institutions resulting in higher scores compared to working in university hospitals. Private hospitals, under pressure to keep patient satisfaction at the highest level, seemed to have an advantage in terms of scores in this regard against state and university hospitals with lower satisfaction expectations. The high rate of healthcare professionals working in private hospitals receiving patient rights training may have also increased this score. Spatial design in ICUs can be mentioned as a feature to bring training and research hospitals into alignment with private hospitals. In the literature, some studies have shown and emphasized that a sense of privacy and dignity is more developed in patients hospitalized in single beds and private rooms.^[41,42]^ In recent years, training and research hospitals in Turkey have changed physically and administratively under the name of “city hospital.” The fact that the beds in ICUs in these hospitals are in separate rooms^[43]^ may be the reason for the higher consciousness scores among the nurses. From this point of view, the relationship between privacy awareness and spatial design may be the subject of further research.

Patient rights are a subset of human rights.^[44]^ It has been observed that receiving patient rights education creates a statistically significant difference in the PAS scores of nurses. In the present study, participants who received patient rights training achieved a higher score than those who did not. However, this difference was not found in the physician group but rather in the nurse group. This result revealed that there is a stronger relationship between nurses and patient rights training compared to that indicated in a previous study.^[45]^ Our study also showed that nurses received training on patient rights at a much higher rate than physicians did. The fact that the nurses working in university hospitals in our sample constituted the group with the least training on patient rights partially explains the low PAS scores for this group.

Although there is no standard patient rights training program in Turkey, it is clear that training has affected the PAS scores. In this context, the analysis of the curriculum in the training program and the strengthening of the content that will increase privacy awareness can be assumed further to increase the PAS scores of healthcare professionals.

### 4.2. The need for in-service privacy training

Most healthcare professionals in our study emphasized the need for in-service privacy training. Nurses expressed the need for more training than physicians and AHPs. It has been stated in our study that as the age of the participant increases, there is less need for in-service training. Therefore, it can be said that such in-service training should be given to younger people or that it would be meaningful to include an individual at an early age. The fact that there was a great need for in-service training between the sixth and eleventh working years, and in these years, the profession importance became more aware, shows that the data is meaningful.

The need for in-service privacy training was affected by whether patient rights training was also to be received. Based on the frequency of responses, those who received patient rights training required more in-service training than those who did not. This indicates that previous education is beneficial and strengthens the belief that other education in a similar field can also be of benefit. On the other hand, the rate of indecision about in-service privacy training was higher for those who had not received patient rights training than those who had received training. This result could be based on the question only assessing the need for training, while the training content is not mentioned. Further research can examine the expectations of in-service privacy training and its content.

## 5. Conclusion

This study found that nurses were the group with the highest PAS scores among healthcare professionals. In addition, the PAS scores of nurses working in private and training and research hospitals were higher than those of other hospital employees. On the other hand, the lowest scores belonged to university hospitals, and receiving patient rights education increased the PAS score of the nurses. However, given that it has been determined that the PAS score does not change with any variable in terms of physicians, the patient rights education method should be questioned, and further research is needed regarding whether physical facilities are directly related. Furthermore, this study showed that all enrolled healthcare professionals required in-service training to gain privacy awareness.

## Acknowledgements

All procedures performed in studies involving human participants were in accordance with the ethical standards of the institutional and/or national research committee and with the 1964 Helsinki declaration and its later amendments or comparable ethical standards. Informed consent was obtained from the participants for publication of this clinical trial details. This manuscript is edited and revised for clarity, consistency, and correctness according to the requirements and guidelines by Scribendi (ESL Academic Editing Service, https://www.scribendi.com, Order # 909751).

## Author contributions

**Conceptualization:** Ahmet Ozdinc, Ahmet Selim Ozkan, Huseyin Bakir, Sedat Akbas.

**Data curation:** Ahmet Ozdinc, Muhittin Calim.

**Formal analysis:** Ahmet Ozdinc, Zuleyha Aydin, Sedat Akbas.

**Funding acquisition:** Ahmet Ozdinc, Huseyin Bakir.

**Investigation:** Ahmet Ozdinc, Ahmet Selim Ozkan, Huseyin Bakir, Sedat Akbas.

**Methodology:** Ahmet Ozdinc, Zuleyha Aydin, Muhittin Calim, Ahmet Selim Ozkan, Huseyin Bakir, Sedat Akbas.

**Project administration:** Ahmet Ozdinc, Muhittin Calim, Ahmet Selim Ozkan, Sedat Akbas.

**Resources:** Ahmet Ozdinc, Sedat Akbas.

**Software:** Ahmet Ozdinc, Zuleyha Aydin.

**Supervision:** Ahmet Ozdinc, Huseyin Bakir, Sedat Akbas.

**Validation:** Ahmet Ozdinc, Muhittin Calim, Huseyin Bakir, Sedat Akbas.

**Visualization:** Ahmet Ozdinc, Muhittin Calim, Sedat Akbas.

**Writing – original draft:** Ahmet Ozdinc, Sedat Akbas.

**Writing – review & editing:** Ahmet Ozdinc, Zuleyha Aydin, Ahmet Selim Ozkan, Sedat Akbas.
